# Current Management of Chronic Myeloid Leukemia with Tyrosine Kinase Inhibitors

**DOI:** 10.4274/Tjh.2013.0108

**Published:** 2013-09-05

**Authors:** İbrahim C. Haznedaroğlu

**Affiliations:** 1 Hacettepe University Medical School, Department of Hematology, Ankara, Turkey

**Keywords:** Chronic myeloid leukemia, Tyrosine kinase inhibitor, Imatinib, Nilotinib, Dasatinib, Bosutinib, Ponatinib

## Abstract

The clinical outcomes and survival of tyrosine kinase inhibitor (TKI)-treated patients with chronic myeloid leukemia (CML) have been significantly improved. The aim of this editorial is to outline critical steps of TKI administration practices during the long-term clinical course of CML based on data obtained from randomized clinical trials and international recommendations. The efficacy of TKI treatment, TKI side effects, off-target complications, and long-term morbidities due to both the disease and the drug are common arguments in the management of CML. Complete hematological response, early complete cytogenetic response, faster major molecular response, and deeper, more durable molecular responses (MR4, MR4.5, MR5) are the ultimate goals for TKI-receiving patients with CML.

**Conflict of interest:**None declared.

## Abstract (tr)

Kronik myeloid lösemi (KML) tedavisinde tirozin kinaz inhibitörü (TKI) grubu ilaçların kullanımı klinik gidişi ve hastaların yaşam süresini son derece olumlu etkilemiştir. Bu yazının amacı, KML’nin uzun klinik seyri boyunca yapılan TKI uygulamalarının kritik basamaklarını randomize klinik çalışma verileri ve uluslararası rehberler ışığında irdelemektir. KML yönetiminde TKI tedavisinin etkinliği, TKI yan etkileri, ilaçların hedef-dışı komplikasyonları, ve hem hastalık seyrine hem de uygulanan ilaçlara bağlı uzun dönem morbiditeler önemli tartışma noktalarını teşkil etmektedir. Tam hematolojik yanıt, erken tam sitogenetik yanıt, daha çabuk gelişen, daha derin (MR4, MR4.5, MR5), ve dayanıklı major moleküler yanıtlar TKI tedavisi alan KML hastalarında klinik seyir boyunca ulaşılması gereken hedefler konumundadırlar.

## INTRODUCTION

Current initial frontline therapy for chronic myeloid leukemia (CML) is chronic oral administration of tyrosine kinase inhibitor (TKI) [1,2]. During the last decade, the introduction of TKI to the treatment regimen of CML has significantly affected the survival of patients. Imatinib mesylate was the first TKI in the clinic. The survival benefit of imatinib in CML is excellent [3]. Next-generation TKIs, namely dasatinib [4,5], nilotinib [6], bosutinib [7], and ponatinib [8], were then developed for the management of CML patients. The clinical outcomes and survival of tyrosine kinase inhibitor (TKI)-treated patients with chronic myeloid leukemia (CML) have been significantly improved. The proper clinical and laboratory monitoring of CML patients is absolutely necessary to reach those successful outcomes [9,10,11]. Complete hematological response (CHR), early complete cytogenetic response (CCyR), faster major molecular response (MMR), and deeper, more durable molecular responses (MR4, MR4.5, MR5) are the ultimate goals for TKI-receiving patients with CML [12]. During the era of 3rd generation TKIs, excellent molecular responses are the most important targets in CML. The surrogate markers of CML outcome (rate, depth, and time to cytogenetic and molecular response) are vital in the clinical management of the disease [1,13,14,15,16,17]. 

Critical evaluations of CML patients to hit those targets should be made at the baseline and at the 3rd, 6th, 12th, 18th, and 24th months after TKI administration. The treatment milestones are checked during the time-line of evaluation [12]. Therapeutic expectations have increased in the field of CML. The functional cure of the disease is now possible with TKIs. Likewise, the molecular responses of MR4 or MR4.5 could lead to the discontinuation of the drug with proper molecular monitoring (TFR; treatment-free remission) within the context of clinical trials [18]. On the other hand, disease progression (accelerated phase (AP) CML or blastic crisis (BC)) under TKI is a great disaster [19,20,21]. Survival after progression into AP/BC is still significantly shorter, even in the TKI era. However, the risk of progression has been decreased with the introduction of more powerful TKIs [22,23,24]. Major attention should be given for the prevention of disease progression, particularly for the treatment-nave CML or TKI-refractory diseases. Clinical response, the depth of response, and the impact of TKI use on the disease outcome should always be the focuses during the long-term management of CML [12]. 

Investigational efforts tried to improve the results of CML first-line therapy of imatinib obtained from the International Randomized Study of Interferon and STI571 trials. Those attempts included imatinib dose increase, particularly in high-Sokal risk patients [25]; imatinib-based combinations [26]; and the setting of second-generation TKIs as first-line therapy [24,27]. Dose optimization studies of TKI, such as the German CML IV [28] and TIDEL [29], have been taken into account for increments in safety, efficacy, tolerability, adherence, and acceptably manageable drug toxicity. The aim of this review is to outline critical steps of TKI administration practices during the long-term clinical course of CML based on data obtained from randomized clinical trials (RCTs) and international recommendations. The efficacy of TKI treatment, TKI side effects, off-target complications, and long-term morbidities due to both the disease and the drug are common arguments in the management of CML. Standardized definitions of molecular response in CML under TKI have been given by the European LeukemiaNet (ELN). MR4 is achieved with a BCR-ABL expression of <0.01%, MR4.5 with <0.0032% BCR-ABLIS, and MR5 with <0.001% BCR-ABLIS [28]. 

**Baseline Evaluation and Management of the Patient with CML**

Standard baseline evaluation of the de novo CML patient includes exact medical diagnosis of CML, basic laboratory evaluation covering complete blood count (CBC) [30] and peripheral blood smear (PBS), bone marrow histopathology, conventional cytogenetics and/or FISH analyses for the Ph* chromosome, and quantitative molecular analyses for BCR-ABL1. Tumor load and disease phase should be defined [12]. Newly diagnosed chronic-phase CML patients should be stratified based on the Sokal [31], Euro/Hasford [32], and EUTOS [33] CML prognostic scoring systems. Novel recent investigations for de novo CML patients have examined the validity of gene expression profiling, genetic polymorphisms, next-generation genomics, multidrug resistance genes (MDR, OCT1), fusion transcripts, and preexisting BCR-ABL kinase domain mutations [34,35,36,37,38,39,40,41,42,43]. 

Current initial TKI treatment for chronic-phase CML is imatinib at 400 mg p.o. [12]. Second-generation TKIs, namely dasatinib at 100 mg p.o. [27] and nilotinib at 600 mg p.o. [24], have also been registered for the first-line therapy of CML. There is a tendency for the prescription of more powerful TKIs in high-Sokal risk CML patients and high-risk patients with complex karyotypic abnormalities at the beginning of the disease for the prevention of disease progression. Likewise, young and low prognostic risk CML patients are candidates for second-generation TKIs for the sake of drug discontinuation in the future. However, heterogeneous presentation and course of CML, individual characteristics, compliance and preferences of the patients, comorbidities, different toxicity profiles of the drugs, and the physician-clinical center experience must all be considered during the initial decision making for first-line TKI usage in newly diagnosed chronic-phase CML [12,24,27]. 

**Evaluation and Management at the 3rd Month after the Initiation of TKI in the Patient with CML **

Standard disease assessments at the 3rd month of oral TKI administration for the chronic-phase CML patient include critical clinical evaluation and CBC/PBS to reveal CHR, cytogenetic analyses to evaluate the cytogenetic response, and quantitative molecular BCR-ABL analyses to identify molecular response [12]. Optimal response at the 3rd month of imatinib administration is CHR and minor cytogenetic response. However, particularly after the introduction of the powerful second-generation TKIs, namely nilotinib and dasatinib, to the first-line therapy of CML, the expectations in response become higher. Recent RCT studies [24,27,44,45,46,47,48,49,50] indicated that the critical BCR-ABL transcript level (10% cut-off value) the 3rd month following the start of TKI treatment may have prognostic significance in patients with CML. This scientific observation has been made with imatinib in GIMEMA [44], German CML IV [26], Hammersmith [51], DASISION [52], and ENESTnd [22] trials, and with dasatinib in DASISION [49] and with nilotinib in ENESTnd [22] trials. Challenges for the widespread routine use of the 10% BCR-ABL transcript cut-off at the 3rd month of TKI are present. First, the estimated ratio of BCR-ABL/ABL is highly technique-dependant. Many laboratories in the world are still not qualified for the international harmonization of scale (IS). High ratio values on the IS scale, house-keeping control gene problems, variations in samples, delays in the exact molecular assessment time after TKI administration, and early unexpected variation kinetics of response in indivi dual CML patients complicate the universal application of the 10% BCR-ABL transcript cut-off at the 3rd month of TKI. Furthermore, the tumor burden at diagnosis, prognostic scoring, gene profile, cytoreduction with TKI dosage, treatment adherence, and numerous confounding effects may obscure the real-life picture at the 3rd month of TKI usage outside clinical trials. Nevertheless, any CML patient that does have a BCR-ABL over 10% after 3 months of TKI presents a strong warning, requiring more careful and more frequent monitoring based on the clear RCT data. If the CML patient exhibits no CHR and/or no minor cytogenetic response, the failure of the first-line TKI is evident. If the initial failed TKI treatment for CML was imatinib, then nilotinib or dasatinib should be given. If 1 of the 2 second-generation TKIs (nilotinib or dasatinib) was used as the first-line therapy and failed, the other (dasatinib or nilotinib) could be administered particularly based on the mutation data. During the treatment decision for 2nd line TKIs, a mutational analysis shall be performed. Increasing the dose of imatinib has been tried in the literature but seems to be a dying practice in the era of stronger TKIs. Drug tolerability and adherence to the treatment should always be sought. Effective management of the treatment-related adverse effects is a vital part of the CML care [12]. 

**Evaluation and Management at the 6th Month after the Initiation of TKI in the Patient with CML **

Standard disease assessments at the 6th month of oral TKI administration for the chronic-phase CML patient include critical clinical evaluation to establish CHR, cytogenetic analyses to evaluate the cytogenetic response, and quantitative molecular BCR-ABL analyses to identify molecular response. Optimal response at the 6th month of imatinib is at least partial cytogenetic response (Ph* chromosome lower than 35%) [12]. However, particularly after the introduction of the powerful second-generation TKIs, namely nilotinib and dasatinib, to the first-line therapy of CML, the expectations in response become higher. CCyR at 6 months and/or BCR-ABL below 1% following 6 months of second-generation TKIs are considered as optimal. Any CML patient that does have a BCR-ABL over 10% and/or Ph* chromosome over 35% after 6 months of TKI treatment (particularly nilotinib and dasatinib) may be accepted as a failed case and the treatment strategy may be changed. Those higher treatment milestones could be applied to the first-line imatinib-receiving CML patients and a switch to second-generation TKIs may be performed. Cumulative incidence of MMR is higher with both nilotinib and dasatinib. An early switch from imatinib to a second-generation TKI is rational since RCTs have indicated the higher probability of obtaining better responses as well as progression-free survival and overall survival [24,27]. Prevention of disease progression seems to be better achieved with more powerful second-generation TKIs. Specific long-term drug adverse effects (such as pleuropulmonary syndrome for dasatinib and metabolic syndrome for nilotinib), as well as increased treatment costs, should be considered. Drug tolerability and adherence to the treatment should always be sought [12]. 

**Evaluation and Management at the 12th Month after the Initiation of TKI in the Patient with CML**

Standard disease assessments at the 12th month of oral TKI administration for the chronic-phase CML patient include critical clinical evaluation to establish CHR, cytogenetic analyses to examine the cytogenetic response, and quantitative molecular BCR-ABL analyses to identify molecular response [12]. Additional karyotypic abnormalities should be searched in the BM cytogenetics. Optimal response at the 12th month of imatinib usage is at least CCyR. However, particularly after the introduction of the powerful second-generation TKIs, to the first-line therapy of CML, the expectations in response become higher [1,6,8,15,16,22,28,44,45,48,50,51,53,54,55,56,57,58,59,60,61,62,63]. CCyR at 12 months and BCR-ABL below 0.1% following 6 months of second-generation TKIs are considered as optimal based on international guidelines. Any CML patient that does have a BCR-ABL over 1% and/or Ph* chromosome over 1% after 12 months of TKI usage (particularly nilotinib and dasatinib) may be accepted as a failed case and the treatment strategy may be changed. Those higher treatment milestones could be applied to the first-line imatinib-receiving CML patients and a switch to second-generation TKIs may be performed. Drug tolerability and adherence to the treatment should always be sought [12]. 

**Evaluation and Management at the 18th Month after the Initiation of TKI in the Patient with CML**

Standard disease assessments at the 18th month of oral TKI administration for the chronic-phase CML patient include critical clinical evaluation to establish CHR and CCyR, and quantitative molecular BCR-ABL analyses to identify molecular response [12]. Optimal response at the 18th month of imatinib is at least MMR. However, particularly after the introduction of the powerful second-generation TKIs, namely nilotinib and dasatinib, to the first-line therapy of CML, the expectations in response become higher [1,4,6,10,11,17,23,38,39,42,44,45,47,48,49,50,51,52,53,54,55,56,57]. CCyR at 18 months and BCR-ABL below 0.1% following 18 months of second-generation TKIs are considered as optimal. Any CML patient that does have a BCR-ABL over 1% and/or Ph* chromosome over 1% (absence of CCyR) after 18 months of TKI usage (particularly nilotinib and dasatinib) may be accepted as a failed case and the treatment strategy may be changed [15]. Those higher treatment milestones could be applied to the first-line imatinib-receiving CML patients and a switch to second-generation TKIs may be performed. Drug tolerability and adherence to the treatment should always be sought [12]. 

**Evaluation and Management at the 24th Month and Thereafter Following the Initiation of TKI in the Patient with CML **

Standard disease assessments at the 24th month of oral TKI administration for the chronic-phase CML patient include critical clinical evaluation to establish CHR and CCyR, and quantitative molecular BCR-ABL analyses to identify molecular response [12]. Optimal response at the 24th month of imatinib is at least the continuation of MMR. However, particularly after the introduction of the powerful second-generation TKIs, namely nilotinib and dasatinib, to the first-line therapy of CML, the expectations in response become higher. CCyR at 24 months and BCR-ABL below 0.1% following 24 months of second-generation TKIs are considered as optimal. Any CML patient that does have a BCR-ABL over 1% and/or Ph* chromosome over 1% after 24 months of TKI usage (particularly nilotinib and dasatinib) may be accepted as a failed case and the treatment strategy may be changed [15]. Those higher treatment milestones could be applied to the first-line imatinib-receiving CML patients and a switch to second-generation TKIs may be performed. Drug tolerability and adherence to the treatment should always be sought. Quality of life is especially a matter of concern in CML patients receiving long-term, maybe lifetime, TKI drugs [12]. 

In the case of intolerance to any TKIs and/or multi-TKI-resistant CML cases with or without mutations, third-line treatment includes bosutinib, ponatinib, allogeneic stem cell transplantation, and experimental therapies [64,65,66]. ABL domain mutations leading to the increments in the BCR-ABL oncogenicity may be detected during the TKI therapy. The TKI regimen may be altered with another TKI based on the type of the mutation. Sometimes the entire treatment strategy of CML has to be changed because of the presence of the ABL domain mutation. For instance, T315I is a unique mutation making the CML patient irresponsive to most TKIs (excluding ponatinib) and leads allografting to become an option in the case [15]. Combination treatments such as TKI plus interferon [67] are still a matter of research and are rarely used outside of clinical trials. 

Evaluation for the discontinuation of TKIs in the superior TKI-responder patient with CML should be performed in the long term, for instance after 2 years. Patients with deeper molecular responses (MR4, MR4.5, MR5) are candidates for TKI discontinuation [68]. MR4 is achieved with a BCR-ABL expression of <0.01%, MR4.5 with <0.0032% BCR-ABLIS, and MR5 with <0.001% BCR-ABLIS [28,46]. Treatment-free remissions and re-induction of the remission with the same TKI seem to be possible based on the data from the STIM trial [68]. Pregnancy represents a cause for TKI discontinuation because of the negative impact of any TKI on organogenesis. Patients with AP/BC CML should be treated with the most powerful TKI available and multi-agent chemotherapy before allografting [19,20,21,65,69,70,71]. Since those patients with advanced-phase CML still do have a worse prognosis, prevention of disease progression is the most significant aspect of CML disease management. 

**Future Perspectives of CML **

ELN 2013 recommendations have established how to proceed in clinical decision making in the CML patients receiving TKIs based on the response status (Table 1). 72 The future of CML and TKI treatment will reveal better understandings of the disease pathobiology, leukemic stem cells, signal transduction, and their translations to the patient’s care [34,35,36,37,38,39,40,41,42,43]. Cure of CML and the eradication of minimal residual disease via the multi-hit drugs with distinct biological actions would be possible. The cessation of therapy with the aim of cure, stem cell depletion, stem cell exhaustion, and immunological control of the disease may be the future strategies in the management of CML. 

## CONFLICT OF INTEREST STATEMENT

The authors of this paper have no conflicts of interest, including specific financial interests, relationships, and/ or affiliations relevant to the subject matter or materials included. 

## Figures and Tables

**Table 1:**
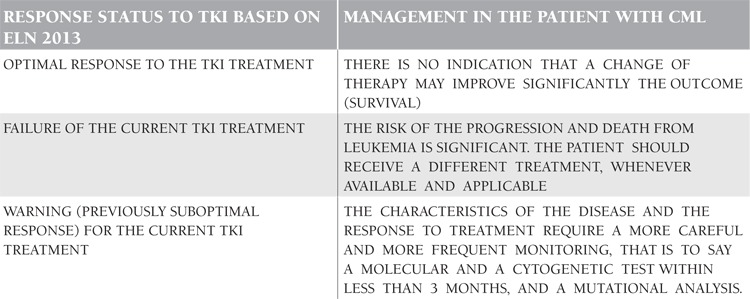
European LeukemiaNet (ELN) 2013 recommendations for the clinical decision making in the chronic myeloid leukemia (CM)L patients receiving tyrosine kinase inhibitor (TKI) based on the response status during the follow-up and monitoring
